# Supplementary material for: STatistical Inference Relief (STIR) feature selection
Source: Bioinformatics. 2018 Sep 18;35(8):1358–65. doi: 10.1093/bioinformatics/bty788 (PMC6477983; doi:10.1093/bioinformatics/bty788)
Supplement: Supplementary Data [file bty788_supplementary_data.pdf]

Supplementary material:
Statistical Inference Relief (STIR) feature selection

Trang T. Lê[1], Ryan J. Urbanowicz[1], Jason H. Moore[1], and Brett A. McKinney[2]

[1] *Institute for Biomedical Informatics, University of Pennsylvania, Philadelphia, PA 19104*
[2] *Tandy School of Computer Science, University of Tulsa, Tulsa, Oklahoma 74104*
[2] *Department of Mathematics, University of Tulsa, Tulsa, Oklahoma 74104*

1 Supplement 1: STIR scores versus the original difference from Relief-based scores

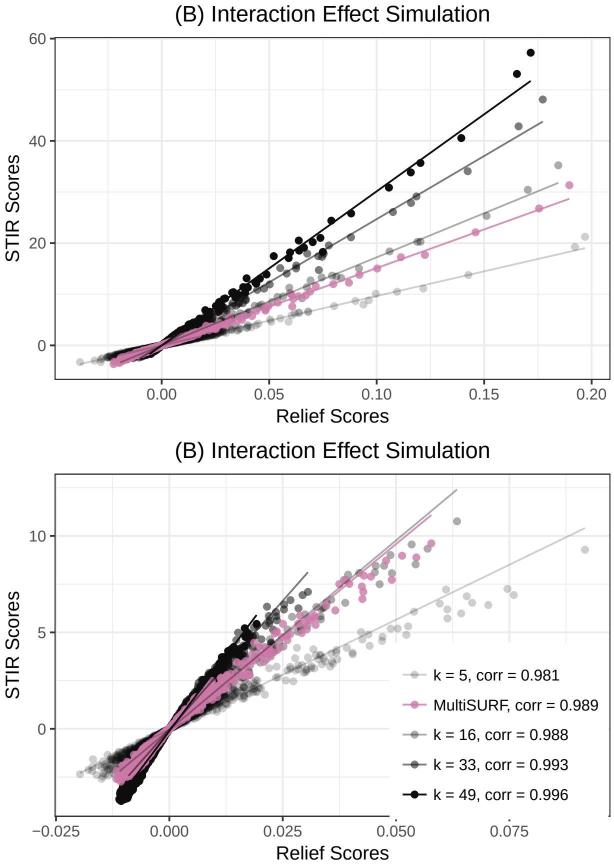

Figure 1: Correlation values of STIR pseudo t-statistic and the original Relief-based scores (diff function) are above 0.98.

2 Supplement 2: RNA-Seq analysis of major depressive disorder genes.

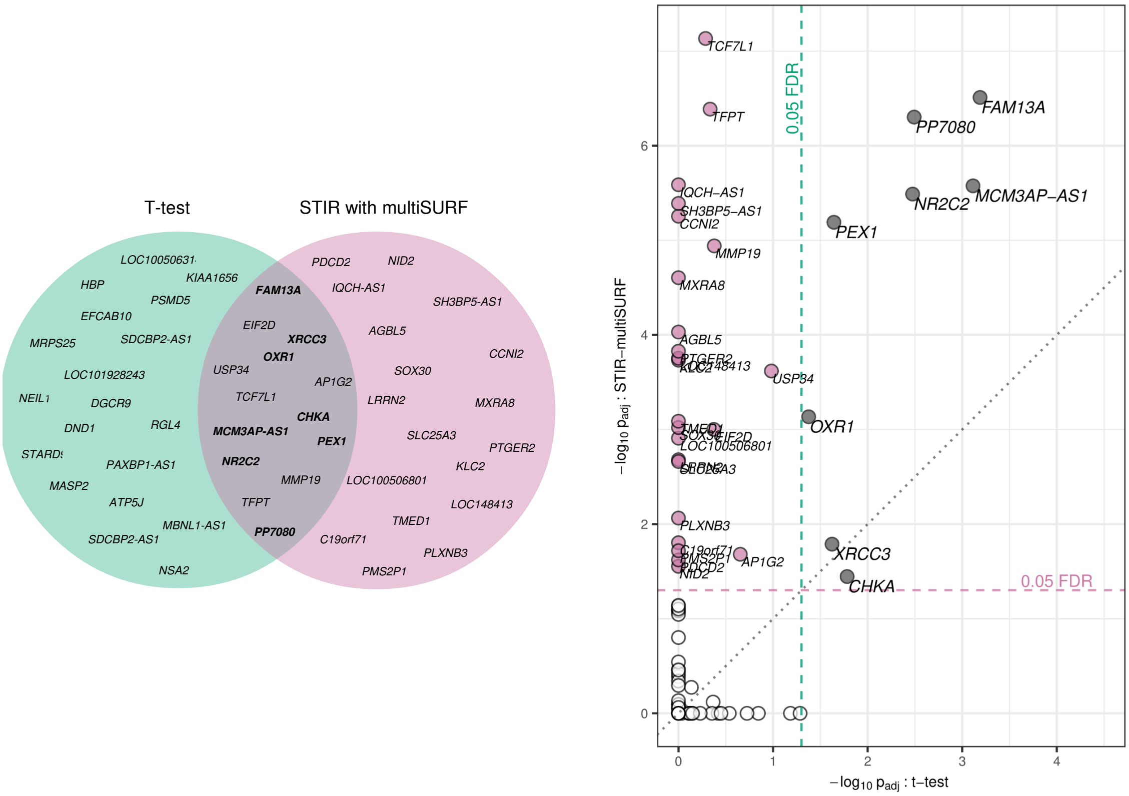

Figure 2: 32 top genes that are most associated with major depressive disorder found by STIR-multiSURF (mauve set) and standard t-tests (green set) from RNA-Seq analysis. 32 STIR-multiSURF genes (mauve set) are significant at the FDR-adjusted 0.05 level. Of the top 32 most significant t-test genes listed, eight are found statistically significant by STIR-multiSURF (**bold**). STIR identifies these significant main effects and additional candidate genes.
